# Reattachment of Coronal Tooth Fragment: Regaining Back to Normal

**DOI:** 10.1155/2013/286186

**Published:** 2013-04-22

**Authors:** B. Vishwanath, Umrana Faizudin, M. Jayadev, Sushma Shravani

**Affiliations:** Department of Conservative Dentistry & Endodontics, Panineeya Dental College, Hyderabad 60, India

## Abstract

Dental trauma is such a situation wherein the patient is affected both socially and psychologically. During their first dental visit, these patients with trauma are in pain and need emergency treatment. Such patients are quite apprehensive because of impaired functions, esthetics, and phonetics. The prime objective while handling such cases is successful pain management with immediate restoration of function, esthetics, and phonetics. The advances in adhesive dentistry have allowed dentists to use the patient's own fragment to restore the fractured tooth. Reattachment is such an ultraconservative technique which provides safe, fast, and esthetically pleasing results. This paper discusses fragment reattachment technique and presents a clinical case of complicated crown fracture.

## 1. Introduction 

Traumatic tooth fractures are the common reason for seeking dental care. Most dental injuries occur between 2 and 3 years and between 8 and 12 years of age; they are more common in boys than in girls because of their active involvement in extracurricular activities [1–3]. The most frequent causes of trauma are falls; bicycle, motorcycle, and car accidents; sports activities; collision with other people and objects; and domestic violence fights and physical assault [4, 5]. Prevalence of trauma to maxillary incisors accounts for about 37%; this is because of their anterior positioning and protrusion caused by the eruptive pattern [6, 7]. Coronal fracture is the frequent type of dental trauma in the permanent dentition [8, 9]. Eighty percent of traumatized incisors have fracture line proceeding in an oblique direction from labial to lingual aspect [7, 10].

Anterior teeth trauma of a young patient is a tragic experience, which requires immediate attention not only because of damage to dentition but also because of the psychological impact it may have on the patient and parents. Various methods and techniques were employed to restore fractured teeth which include pin retained resin, orthodontic bands, stainless steel crowns, porcelain jacket crowns, and complex ceramic restorations [11, 12]. However all these restorations require significant tooth preparation and were not esthetically adequate; moreover they cannot be used in an emergency esthetic situation [13, 14].

The first case report on reattachment of a fractured incisor fragment was published by Chosack and Eidelman in 1964 in which the complicated tooth fracture was managed by endodontic therapy followed by a cast post and core [15]. The use of acid etch technique for the reattachment of fractured fragment was first reported by Tennery [6]. Similar cases were also reported by Starkey [16] and Simonsen [8]. The success of reattachment depends on certain factors like the site of fracture, size of fractured remnants, periodontal status, pulpal involvement, maturity of the root formation, biological width invasion, occlusion, time material used for reattachment, use of post, and prognosis [17]. Reattachment is a way to restore the natural shape, contour, translucency, surface texture, occlusal alignment, and color of the fragment along with a positive emotional and social response from the patient to the preservation of natural tooth structure, and it is also an economical and a conservative procedure [8, 18–23].

## 2. Case Report

**Figure 1:**
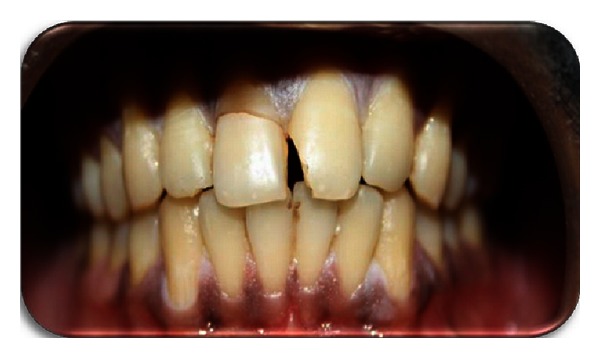
Preoperative photograph.

A 23-year-old male patient reported to the Department of Conservative Dentistry and Endodontics, Panineeya Mahavidyalaya Institute Of Dental Sciences And Research Centre, Hyderabad, India, with the chief complaint of broken upper front tooth following trauma three days ago which occurred due to a motorcycle accident (Figure 1).

Clinical examination revealed horizontal fracture in the middle third region of the right maxillary incisor involving enamel and dentin with exposure of the pulp and the fractured fragment being loosely attached to the tooth. The fracture was not evident palatally. Left maxillary incisor showed mesioangular incisal chipping. Soft tissue examination showed laceration of the upper lip.

**Figure 2:**
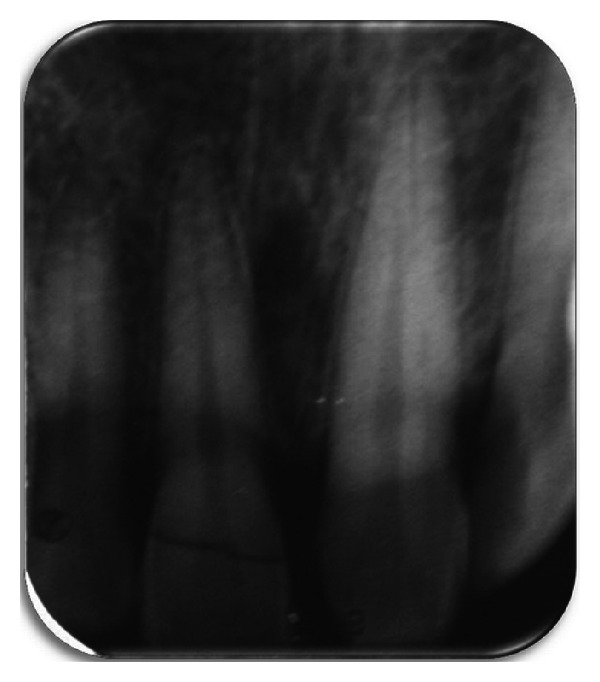
Preoperative radiograph.

A periapical radiographic examination revealed an oblique fracture labiopalatally; the root formation was complete with no extrusion of the tooth (Figure 2). The patient expressed the desire to maintain the tooth and restore it, as it is economical compared to an indirect restoration. A detailed explanation about the treatment plan was given to the patient, which included endodontic treatment, and then reattachment of the tooth crown using a fiber post and informed consent is taken from the patient.

**Figure 3:**
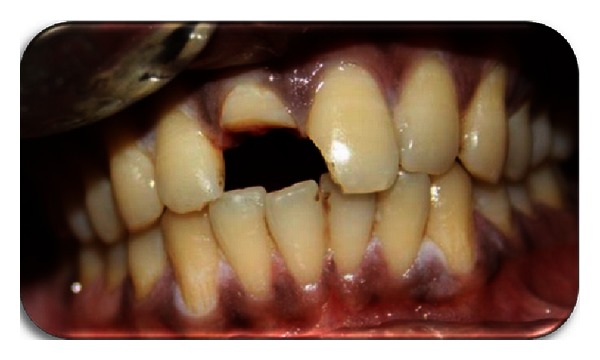
After fragment removal.

**Figure 4:**
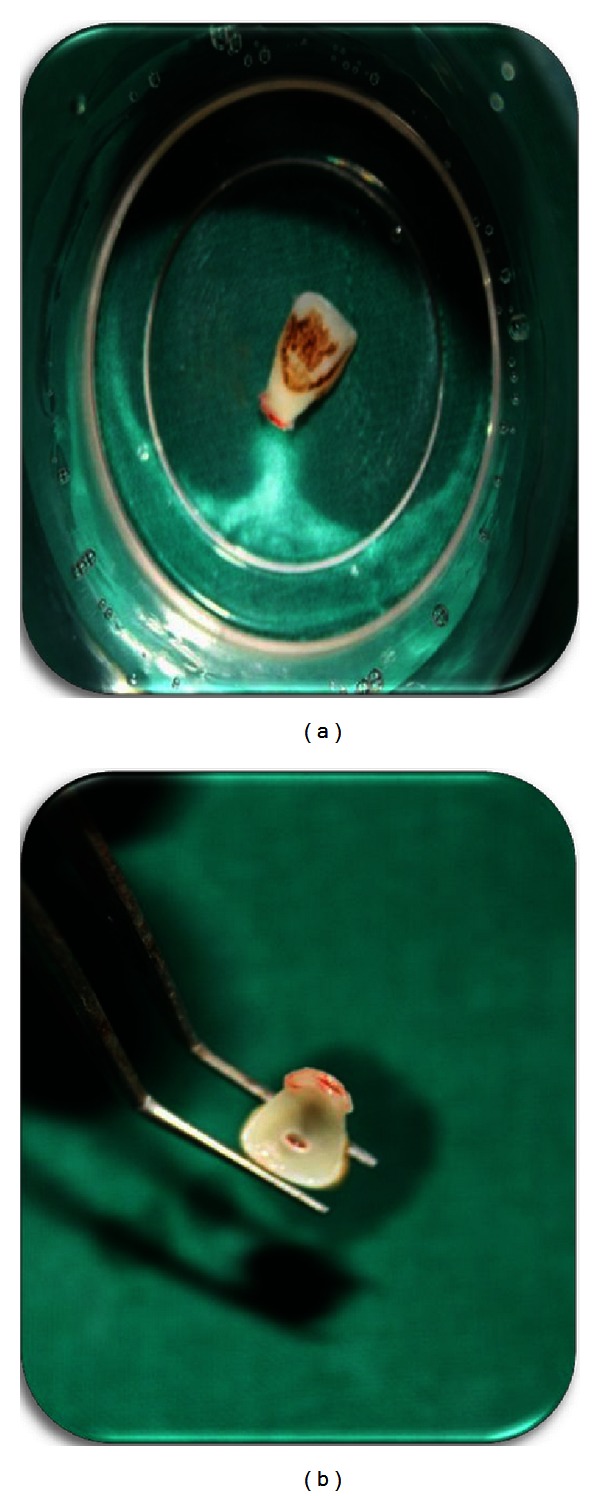
Fracture fragment.

**Figure 5:**
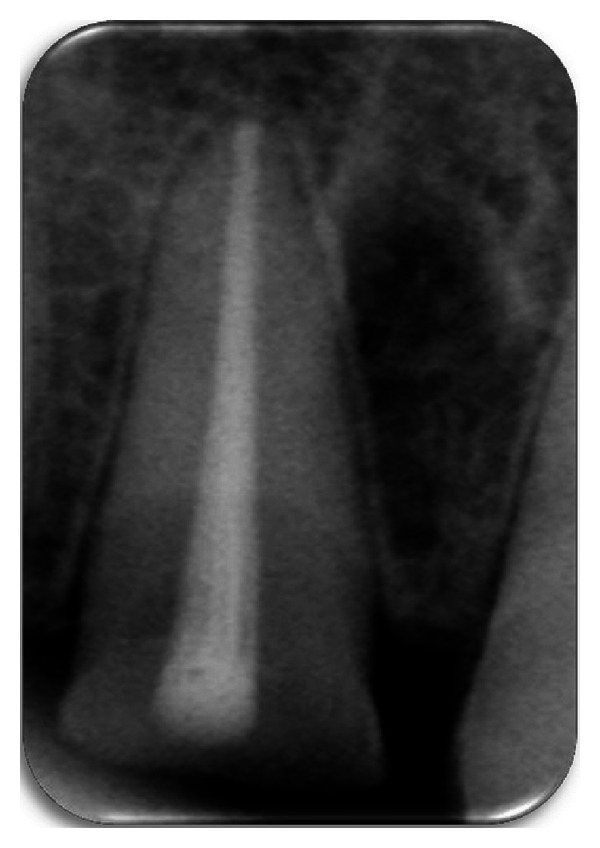
Obturation.

**Figure 6:**
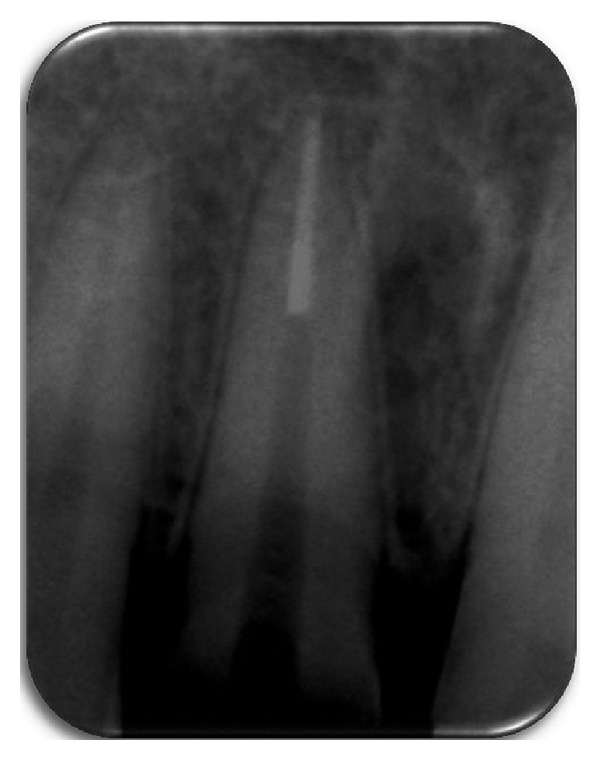
Post space preparation.

**Figure 7:**
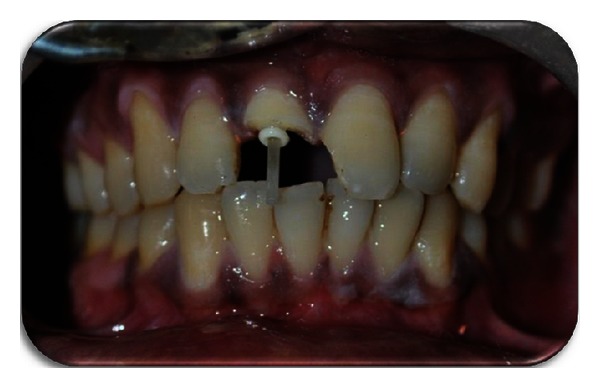
Post placement.

**Figure 8:**
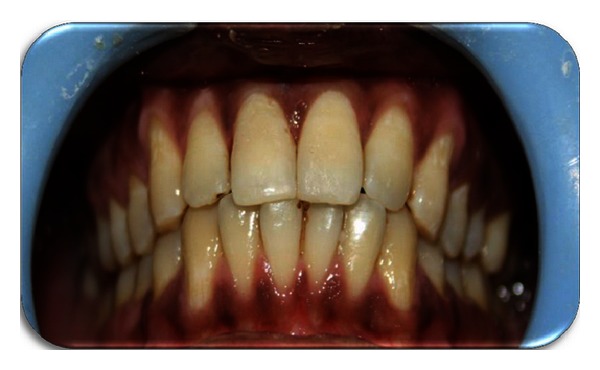
Postoperative photograph.

Local anesthesia was administered followed by the removal of the fractured segment completely and preserved in physiological saline solution in order to prevent dehydration and discoloration of the tooth fragment (Figures 3 and 4). Following a detailed examination, the fit of the fragment was checked. Working length was established with the help of radiograph followed by the biomechanical preparation by step back technique, with the master file being 45 k-file. Irrigants like 2.5% sodium hypochlorite and saline solution were used during the preparation alternately. The root canal was dried with paper points and obturated using lateral condensation technique with gutta percha (Dentply Maillefer, Ballaigues, Switzerland) and AH plus sealer (Maillefer, Dentply, Konstanz, Germany) (Figure 5). After completion of the endodontic treatment, the root canal was prepared for the post placement by removing the gutta percha from the coronal two-thirds of the canal with peeso reamers (drill size 2) (Figure 6). Bevels are placed on the tooth and the fractured fragment, in order to enhance the retention. The fibre post (Dentply Tulsa, Johnson city, US) was tried in the canal and adjusted to the desired length (Figure 7). Space was also prepared in the pulp chamber of the fractured crown fragments for receiving the coronal portion of the post and also the core. The alignment of the coronal fragment was verified with the post in situ. The root canal was then etched with 37% ortho phosphoric acid, rinsed, and blot-dried with paper points, and bonding agent was applied. The post was then luted in the canal using dual cured resin luting cement (Ivoclar Vivadent). The inner portion of the coronal fragment was similarly etched and bonded to the tooth using flowable composite resin (Ivoclar Vivadent) after proper shade matching. The tooth was polished with polishing disc (Figure 8).

Occlusion was verified and postoperative instructions are given to the patient in order to prevent any loading of the anterior teeth. Clinical and radiographic examinations were carried out after 1 month, 3 months, and 6 months and the tooth responded favorably.

## 3. Discussion

Studies have shown that one out of every four persons under the age of 18 will sustain a traumatic anterior crown fracture [24, 25]. Whenever the fracture fragment is available reattachment should be the first choice of treatment [26, 27]. In recent years due to remarkable advancements of adhesive systems and resin composites, it is now possible to achieve excellent results with reattachment of tooth fragments provided that the biological factors, materials, and techniques are logically assessed and managed [28]. As with the conventional restoration, restorative success depends on proper case selection, strict adherence to sound principles of periodontal and endodontic therapies, and the techniques and materials for modern adhesive dentistry [29–31]

In the present case of complicated crown fracture requiring endodontic therapy, the fractured fragment was available and reattachment of the fragment with fiber post is performed. The use of the natural tooth substance offers a conservative, esthetic, and economical option that provides good and long lasting esthetics, restores function, results in a positive psychological response, and is certainly a simple procedure. Adhesive post is used as it has the potential for increased retention, is more flexible, and has modulus of elasticity approximately same as dentin, and when bonded with resin cement it distributes forces evenly along the root [32].

The most common complication of post and core system is debonding [33]; another reason for failure is root fracture [34]. Restoration with cast metal posts can cause wedging forces coronally that may result in irreversible failure because of fracture of an already weakened root [35]. Whereas fiber-reinforced composite resin post has demonstrated negligible root fracture. Studies have indicated that dentin-bonded resin post-core restorations provide significantly less resistance to failure than cemented custom cast posts and cores [36, 37]. In addition, the fiber-reinforced posts are used with minimal preparation because it uses the undercuts and surface irregularities to increase the surface area for bonding, thus reducing the possibility of tooth fracture during function or traumatic injury [38].

The clinician must consider that a dry and clean working field and proper use of bonding protocols and bonding materials are the key to achieve success in adhesive dentistry. Reattachment failures occur as a result of new trauma or parafunctional habits, so fabrication of a mouth guard and patient education about treatment limitations enhance clinical success [39].

With all traumatic injuries, followup is of critical importance and the patient should be followed for 3, 6, and 12 months and yearly for 5 years [40]. At these follow-up visits esthetics, tooth mobility, and periodontal status should be confirmed both clinically and radiographically.

## 4. Conclusion

Because of larger incidence of trauma to dental tissues and their supporting structures, it is important to have proper knowledge of the techniques available and their indications, along with risk benefit ratio. The reattachment of the tooth fragment is possible only when the fragment is available and can be improved with different adhesive techniques and restorative materials. The main concern is to educate the population to preserve the fractured fragment and seek immediate dental care.
